# The complete chloroplast genome of *Mitrasacme pygmaea* (Loganiaceae)

**DOI:** 10.1080/23802359.2020.1772686

**Published:** 2020-06-12

**Authors:** Xiaolang Du, Lan Cao, Guoyue Zhong, Zejing Mu

**Affiliations:** Research Center for Traditional Chinese Medicine Resources and Ethnic Minority Medicine, Jiangxi university of Traditional Chinese Medicine, Nanchang, China

**Keywords:** Complete chloroplast genome, *Mitrasacme pygmaea*, Loganiaceae

## Abstract

The complete chloroplast genome of *Mitrasacme pygmaea* was sequenced and assembled for the first time. The chloroplast genome is 152,611 bp in length, containing a large single-copy (LSC) region of 83,881 bp and a small single-copy region (SSC) of 18,110 bp, separated by a pair of inverted repeats (IRs) of 25,310 bp. The genome contains 113 unique genes, including 79 protein-coding genes, 30 tRNA genes, and 4 rRNA genes. Among them, 15 genes have one intron each and 3 genes contain two introns. The overall GC content is 37.9%, while the corresponding values of LSC, SSC, and IR regions are 36.0%, 31.7%, and 43.4%, respectively. Phylogenetic analysis showed that *M. pygmaea* is sister to *Gentiana tibetica* and provided new insight into the evolution of Loganiaceae.

*Mitrasacme* Labill., a genus of Loganiaceae (Gentianales) with about 55 species, are mainly distributed in Australia. Only two species of *Mitrasacme*, *M. indica* and *M. pygmaea*, are found in China and widely distributed in the southern provinces (Li and Leeuwenberg 1996; Gibbons et al. 2015). *Mitrasacme pygmaea* contains phenols, alkaloids and flavonoids (Huang et al. 2016). In Chaoshan (Guangdong province, China), it is often used to treat sore throats and coughs, showing great medicinal value (Wang et al. 2019a). In this study, we sequenced and assembled the chloroplast genome of *M. pygmaea* for the first time, which is also the first complete chloroplast genome of Loganiaceae.

Fresh leaves of *M. pygmaea* were collected from Nanchang, Jiangxi, China (GPS: 28°40′30″, 115°44′56.32″). Herbarium voucher (Voucher No. JXCM20190506) is deposited in the Medicinal Herbarium, Jiangxi University of Traditional Chinese Medicine, Nanchang, China. Total genomic DNA was extracted using the modified CTAB method (Doyle and Doyle 1987) and sequenced on an Illumina NovaSeq platform with paired-end reads of 150 bp. The GetOrganelle pipeline (Jin et al. 2018) were carried out for the *de novo* assembly of chloroplast genome. Genes were annotated by PGA (Qu et al. 2019) and visually checked in Geneious v8.0.2 (Kearse et al. 2012) using chloroplast genome of *Catharanthus roseus* (GenBank accession NC_021423) as reference. The predicted transfer RNAs (tRNAs) were confirmed by tRNAscan-SE 2.0 (Lowe and Chan 2016). Finally, the complete chloroplast genome with annotations was submitted to the GenBank (accession MT330399).

The size of complete chloroplast genome of *M. pygmaea* is 152,611 bp with high coverage (mean 1771×). It has a typical quadripartite structure, including a large single-copy (LSC) region of 83,881 bp, a small single-copy region (SSC) of 18,110 bp, and a pair of inverted repeats (IRs) of 25,310 bp. There are 79 protein-coding genes, 30 tRNA genes, and 4 rRNA genes. Among these genes, 15 of them (*atpF*, *ndhA*, *ndhB*, *petB*, *petD*, *rpl2*, *rpl16*, *rpoC1*, *rps16*, *trnA-UGC*, *trnG-UCC*, *trnI-GAU*, *trnK-UUU*, *trnL-UAA* and *trnV-UAC*) are single-intron genes, and three genes (*clpP*, *rps12* and *ycf3*) contain two introns. The overall GC content is 37.9%, while the GC content of LSC, SSC, and IR regions are 36.0%, 31.7%, and 43.4%, respectively.

To identify the phylogenetic relationship of *M. pygmaea* in Gentianales, the phylogenetic tree including *M. pygmaea*, four other Gentianales species, and two outgroups (*Olea exasperate* and *Syringa vulgaris*) of Lamiales were reconstructed using complete chloroplast genomes. The sequences were aligned by MAFFT v7.017 plugin (Katoh et al. 2002) and visually checked in Geneious. Phylogenetic analysis was performed by RAxML v8.2 (Stamatakis 2014) using 1000 replicates of a rapid bootstrap analysis with GTRGAMMAI substitution model. The phylogenetic relationships among all sampled Gentianales species were fully resolved with maximum support (Figure 1). In contrast to previous findings (Li et al. 2019; Wang et al. 2019b), *Catharanthus roseus* (Apocynaceae) is sister to *Gelsemium elegans* (Gelsemiaceae) and then grouped as sister with the clade of *Gentiana tibetica* (Gentianaceae) and *M. pygmaea*. The chloroplast genome obtained in this study could provide essential data to determine the phylogenetic position of Loganiaceae and provide new insight into the evolution of Gentianales.

**Figure 1.**
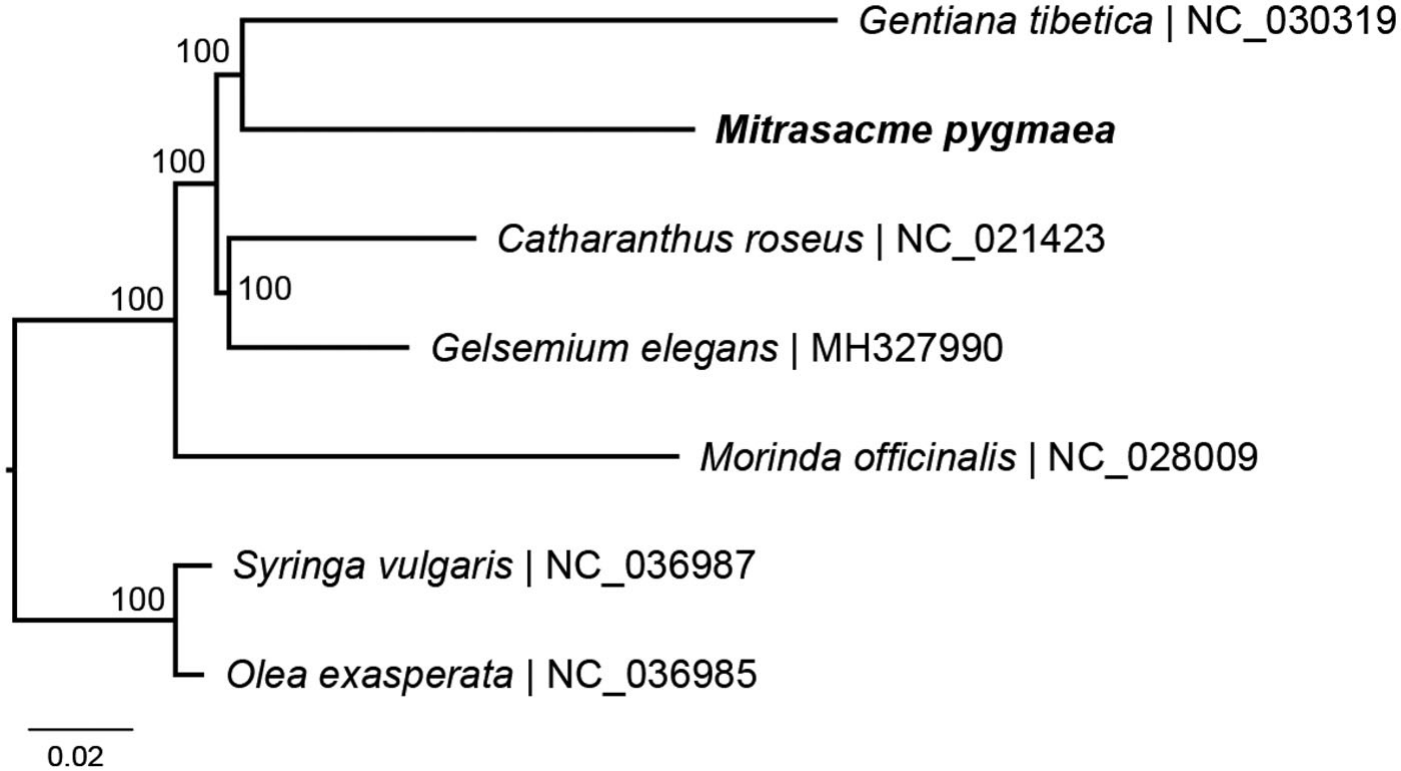
Maximum-likelihood phylogenetic tree based on complete chloroplast genomes. Numbers close to each node are bootstrap support values.

## Data Availability

The data that support the findings of this study are openly available in GenBank at https://www.ncbi.nlm.nih.gov/genbank/, accession numbers [NC_030319, NC_021423, MH327990, NC_028009, NC_036987 and NC_036985]. The complete chloroplast genome generated for this study has been deposited in GenBank with accession number MT330399.
